# Experimental investigation of cylinder liners and performance optimization using ANN

**DOI:** 10.1016/j.mex.2025.103426

**Published:** 2025-06-11

**Authors:** Shekhar T. Shinde, Kishor R. Borole, Kedarnath Chaudhary, Namita Shinde

**Affiliations:** a Bharati Vidyapeeth (Deemed to be University) College of Engineering, Pune 411043, India; b Sinhagad College of Engineering, Pune 411041, India; c SKN College of Engineering, Pune 411041, India; d COEP Technological University, Pune 411005, India

**Keywords:** Cylinder liner, Internal combustion engine, Finite element analysis, Material optimization, Thermomechanical stress, Finite Element Analysis, Artificial Neural Networks

## Abstract

This research investigates the structural integrity and performance optimization of cylinder liners in internal combustion engines, focusing on material selection and thermomechanical stress response. The study aims to enhance efficiency and durability by evaluating the performance of cast iron, nickel-chromium iron alloy, and aluminum alloy under real-world engine conditions. Using Finite Element Analysis, material properties are experimentally assessed to determine thermomechanical stresses, wear characteristics, and heat dissipation behavior. Additionally, Artificial Neural Networks are employed to optimize performance parameters by predicting material behavior under varying thermal and load conditions. Results indicates that

• Nickel-Chromium iron alloy exhibits the lowest combined stress, making it the most suitable material due to superior resistance to thermal stress.
• Unlike cast iron, where thermal-induced stress is too much to bear, aluminum alloy is lightweight but suffers from deformation when it undergoes thermal expansion.
• However, the ANN-based optimization was successful in lowering stress values and allows us to consider future applications as a prediction modelling tool in material selection. The ANN optimization will reduce thermal stress by up to 20 % and is thus a successful method to use with predictive maintenance and improved performance of cylinder liners.

This study shows that using both computational and experimental approaches can allow for optimized performance for engine components and provide future direction for cylinder liner designs that are both stronger and have better performance.

## Graphical abstract

**Figure:**
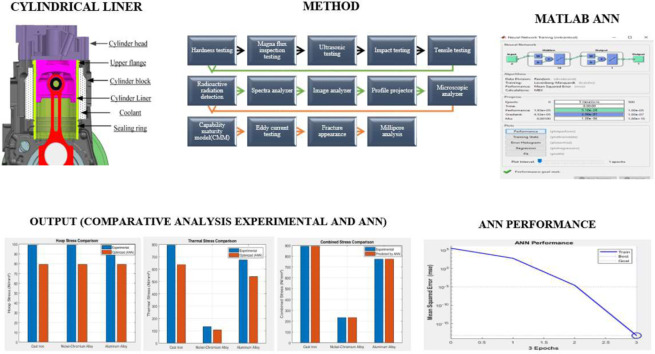

Specifications Table

**Table:** 

Subject area:	Engineering
More specific subject area:	*Internal Combustion Engine*
Name of your method:	*Finite Element Analysis, Artificial Neural Networks*
Name and reference of original method:	
Resource availability:	

## Background

Internal combustion engines are running the show in modern transportation and industrial equipment, and the efficiency and durability of these engines depend on the quality of various components, such as cylinder liners. The liner provides a smooth, wear-resistant surface for the pistons to operate and helps maintain the structural integrity of the engine during severe operational conditions [1]. These liners are designed to tolerate high temperatures, mechanical forces, and friction, while remaining effective for extended periods. The importance of the cylinder undermines wear and tear, optimizes heat transfer, and enhances total engine efficiency [2]. Due to their intrinsic functions, it is necessary to research the materials, failure modes and optimization to improve the longevity and performance of cylinder liners. This research will focus on the materials of cylinder liners, study their performance characteristics, and optimize their performance utilizing Artificial Neural Networks (ANN). Cylinder liners can be either wet or dry. Wet cylinder liners interface with coolant fluids, facilitating heat transfer and thermal management. In the event of overheating, inadequately cooled engine cylinder heads and engine blocks may deform or fail due to thermal stresses [3]. In contrast, dry liners are replaceable separately from the coolant, means they are less likely to leak. However, dry liners have less heat transfer efficiency since the coolant has no direct contact with the liner. Both types of liner still have problems with wear and heat dissipation [4]. Friction and heat which are produced between the piston and the cylinder wall, play a major role in the power loss in engines and therefore it is important to optimize the materials and designs of the liners [5]. Research has shown that cylinder liners produce around 30 % of the friction in the total engine friction and contribute to approximately 5 % of combustion heat loss and 10 % of available power loss due to friction in mechanical systems. Many advances have been made in the understanding of lubrication and materials, however energy loss due to friction is still concerning and there is also a need for continued investigation into optimizing the performance of cylinder liners.

The performance and durability of cylinder liners are directly influenced by the selection of materials used in their production [6]. Typical materials used for cylinder liners include cast irons, alloyed steels, and higher end composite materials however, they offer differing benefits for wear-resistance, thermal conductivity and mechanical strength [7]. The best material choice would have high conductivity in order to dissipate heat, while having sufficient hardness to resist wear and sufficient mechanical properties to be able to sustain the cyclic loads and associated stresses while operating in an engine [8]. This means balancing all required properties for the ideal solution. Thermo-mechanical analysis can be used to help us understand the on of a cylinder liner during normal operational conditions [9]. The liner experiences both expansion and contraction with temperature variations, and together with the continuously dynamic piston, the stresses become highly complex so that liner deformation followed by failure can occur. Thermal expansion mismatch of the liner to the cylinder block can also create cracks, distortions, and in some cases produce catastrophic failures [10]. Through the understanding of temperature gradients and stress distributions, researchers have been able to examine compatibility materials and designs that have minimized thermal fatigue thus potentially improving liner life. This study will examine the cylinder liner in regard to different cylinder liner materials under engine simulated conditions with an analysis of the amount of heat transfer, frictional forces, and the wear resistance of the materials [11]. The study will contribute to improved liner performance and life.

## Method details

A major aim of this work is to study the failing mechanisms of the cylinder liner. The principal failure modes of the cylinder liners are wear, scuffing, cracking and distortion, which can all affect the performance of the engine significantly [12]. Wear is caused by the continual friction between the surface of the liner and piston rings, which leads to material loss and degradation over time [13]. Scuffing, or more severe wear, often results from high local temperatures and inadequate lubrication, which leads to localized welding between the piston and the surface of the liner. Cracking and distortion arise from mechanical stresses and/or thermal expansion mismatches, which can reduce the integrity of the liner and ultimately lead to engine failure [14]. To address these issues, performance optimization approaches based on Artificial Neural Networks (ANN) are considered. ANN-based models can be valuable to predict liner performance from the complex associations of the various factors, e.g., material properties, operating conditions and thermal stresses [15]. Predictive intelligence can be established with ANN models from experimental data, to allow predictions on ideal materials compositions, surface treatment options, as well as forms of lubrication [16]. This enables both researchers and engineers to develop liners with better durability and efficiency while limiting energy loss from friction. The incorporation of ANN in the cylinder liner analysis provides a viable route towards development of advanced engine components, that offer improved performance characteristics [17].

The implications of this study for the automotive and manufacturing industries are extensive. By identifying optimal materials and design alternatives for cylinder liners, this study will lead to the production of more efficient and durable internal combustion engines. Improvements in cylinder liner performance will enable improved fuel efficiency, a reduction in emissions, and a decrease in maintenance costs for both manufacturers and users alike. In addition, the use of ANN-based optimization can pave the way toward a smarter and more data-oriented decision-making process in the design of engine components while allowing for more nuanced performance enhancements designed for particular operational environments. Furthermore, this study lays the groundwork for future research into the and the incorporation of advanced materials, coatings, and surface treatments into cylinder liner designs. Developments such as ceramic coatings, nano-engineered surfaces, and hybrid composite liners could completely revolutionize engine performance by providing better, lower frictional losses and a greater capacity for heat transfer. Together with experimental work, this study provided the basis for ANN-based modelling to optimize cylinder liners and improve engine efficiency overall.

A model for thermomechanical finite element analysis is proposed to determine engine piston stress and fatigue, which includes piston, piston pin, piston ring, bushing, connecting rod and cylinder liner. The proposed model considers contact pressure and oil film on various surfaces in the engine assembly. For establishment of initial clearances, a custom process is implemented which has taken into consideration the piston skirt profile and elastic properties. Of course, dynamic loads are determined using powertrain software to calculate stress and fatigue accurately, therefore, it is assumed these conditions simulate the working conditions of the piston. Ajith Kurian et al. (2022) investigate the effect of honing angle on the tribological behaviour of cylinder liners when boundary lubricated under reciprocating motion. They honed cylinder liner samples to honing angles of 20°, 40°, 60°, 80° or 100° and evaluated them using a linear reciprocating tribometer at two different sliding velocities (0.2 m/s and 0.3 m/s). The results demonstrated that honing angle plays an important role in drags tests friction and wear [19]. Therefore, the 40° honing angle was determined to result in the least coefficient of friction and wear. Their findings offer important information about the honing angle to increase engine efficiency and lessen wear in the liner-ring tribosystem [18].

The study by Ahmad Alshwawra et.al (2020) aims to lower friction in internal combustion engines and was specifically focused on optimizing the piston ring–cylinder liner (PRCL) configuration [19]. The research found that using a liner that is an initial conical and/or ellipsoidal shape and whose deformation in the fired condition causes it to assume a straight and parallel shape a significant amount of friction is reduced. In fact, this study includes numerical simulations that were compared to experimental work and showed that the combined conical and ellipsoidal liner has reduced friction compared to its cylindrical liner counterpart suggesting that there is a major opportunity for the greater reduction of friction through the piston-liner combination. Last but not least, a study by Xinlin Zhong et.al (2023) indicates that in order for internal combustion engines to run in as efficient a manner as possible, the contact between the piston ring and liner, particularly in the dry area follower the Top Dead Centre of the Oil Control Ring must be protected [20]. More significantly, this study was the first of its kind to examine mechanisms of oil distribution in this area. The study utilized experimental techniques and a 3D machine learning model. The authors presented that the vortices downstream of the top ring gap were instrumental in providing oil finally bridging the oil to provide the critical lubrication at the ring-liner interface as it traveled to the liner. Zongyu Yue et al. (2023) studied the critical importance of Internal Combustion (IC) engines in transport and energy (stationary power) applications. The discussion presents ongoing development of IC engines and the possibilities to enhance efficiency and reduce pollution. The importance of IC engines in facilitating future transport and energy systems are emphasized [21]. The editorial discusses directions for research to promote advancement in IC engine and fuel technologies. In addition, the introduction notes 14 technical papers forming a Special Issue, addressing a diverse range of study topics like properties of diesel spray, low and zero-carbon fuel combustion technologies, novel combustion modes, effects of fuel additives, engine performance in extreme environments, and materials and methods of production advancements.

According to Shekhar Shinde et.al (2016) Internal combustion engines are regularly found in many applications within the mechanical industry. For example, they are found in all automobiles, ships, power planes, and power producing units. The cylinder liners of an internal combustion engine are the main wreckers and load bearing components. While the engine is working the cylinder liner is under great distress. All the stresses on the cylinder liner are through heat stress, stress due to the piston and gas pressure [22]. These stresses lead to wear patterns, cylinder liner corrosion, internal or external cracks. All the findings above each affect how cylinder liners operate, as well as decreasing the operating efficiency of the internal combustion engine. As a result, it is important to examine the various causes of liner failure and solutions for strengths and improvements.

### Problem statement

Cylinder liners play a critical role in ensuring the durability and efficiency of an internal combustion engine by providing a wear-resistant surface for the piston to move within the cylinder bore. The makeup of the liner with respect to material, surface finish, and thermal expansion characteristics is a significant contributor to the overall performance, fuel efficiency, and emissions of a Celerio CNG engine. Given the popularity of fuel-efficient and low emission vehicles in today's market, it has become imperative to optimize the performance of the cylinder liners, yet most traditional methods utilize a trial-and-error experimental approach, which is both time-consuming and expensive. Therefore, an approach using an Artificial Neural Network (ANN) provides more than just the opportunity to effectively experiment with the liner, it also allows for predictive modelling of liner performance based on key characteristics including wear rate, thermal conductivity, lubrication efficiency, and combustion qualities.

This study aims to evaluate the mechanical and thermal properties of the cylinder liners in the Celerio CNG engine using experimental methods while incorporating ANN as an optimization of performance. The study will investigate the wear characteristics, temperature trends, and material degradation based on the conditions of use. Using ANN modelling, this study will predict the ideal liner properties of the cylinder to improve efficiency and service life.

### Maruti Suzuki Celerio CNG engine specifications

• **Engine Type:** 1.0 L K10C Dual Jet, Dual VVT (Variable Valve Timing)
• **Fuel Type:** Compressed Natural Gas (CNG) and Petrol (Dual Fuel)
• **Displacement:** 998 mL
• **Maximum Power**
  ○ 59 bhp @ 6000 rpm (CNG)
  ○ 65 bhp @ 6000 rpm (Petrol)
• **Maximum Torque**
  ○ 82.1 Nm @ 3400 rpm (CNG)
  ○ 89 Nm @ 3500 rpm (Petrol)
• **Engine Configuration:** Inline 3-Cylinder
• **Compression Ratio:** 9.5:1
• **Bore x Stroke:** 73.0 mm x 74.0 mm
• **Valvetrain:** DOHC, 12 Valves
• **Cooling Type:** Liquid Cooling
• **Fuel Injection:** Multi-Point Fuel Injection (MPFI)
• **Emission Norm:** BS6

This approach provides a data-driven process for future cylinder liner design enhancements, resulting in improved engine reliability, better fuel economy, and reduced emissions; secondly, it also decreases the reliance on large-scale physical tests.

### Materials selection and characterization

The materials selected for conducting an experimental exploration of cylinder liners include Cast Iron, Nickel-Chromium Iron Alloy, and Aluminium Alloy. The rationale behind the inclusion of these types was established by incorporating their mechanical strength, wear resistance, and thermal conductivity factors. Each of the three materials was investigated for their metallurgical composition, physical properties, and surface attributes to assess their applicability as cylinder liners in the Maruti Suzuki Celerio CNG engine.

The following material properties were studied (Table 1):

**Table 1:** Material properties.

Material	Density (kg/m³)	Young’s Modulus (MPa)	Poisson’s Ratio	Thermal Conductivity (W/mK)	Tensile Strength (MPa)	Yield Strength (MPa)	Fatigue Strength (MPa)
Cast Iron	7100	1,70,000	0.26	60	300	200	150
Nickel-Chromium Iron Alloy	7800	2,10,000	0.3	50	750	500	350
Aluminum Alloy (Al 6061)	2700	69,000	0.33	150	310	276	130

The selected cylinder liner samples were tested for hardness, impact resistance, and microstructure analysis using metallurgical techniques.

## Method

Five-to-seven-cylinder liners were procured for this research project to start the investigation and collect baseline data: A full failure investigation using metallurgical testing was done on the liners using ten different tests to track the failure mode. The primary tests being hardness, magna flux inspection, ultrasonic testing, impact testing, tensile testing, radioactive radiation, and spectral testing to evaluate the material condition. Advanced imaging and metrology methods were performed using an image analyzer, profile projector and microscopic analyzer for surface morphology and structural purposes. Additional testing was done using the capability maturity model (CMM), eddy current testing, fracture appearance and Millipore analysis to fully assess liner condition.

**Fig. 1:**
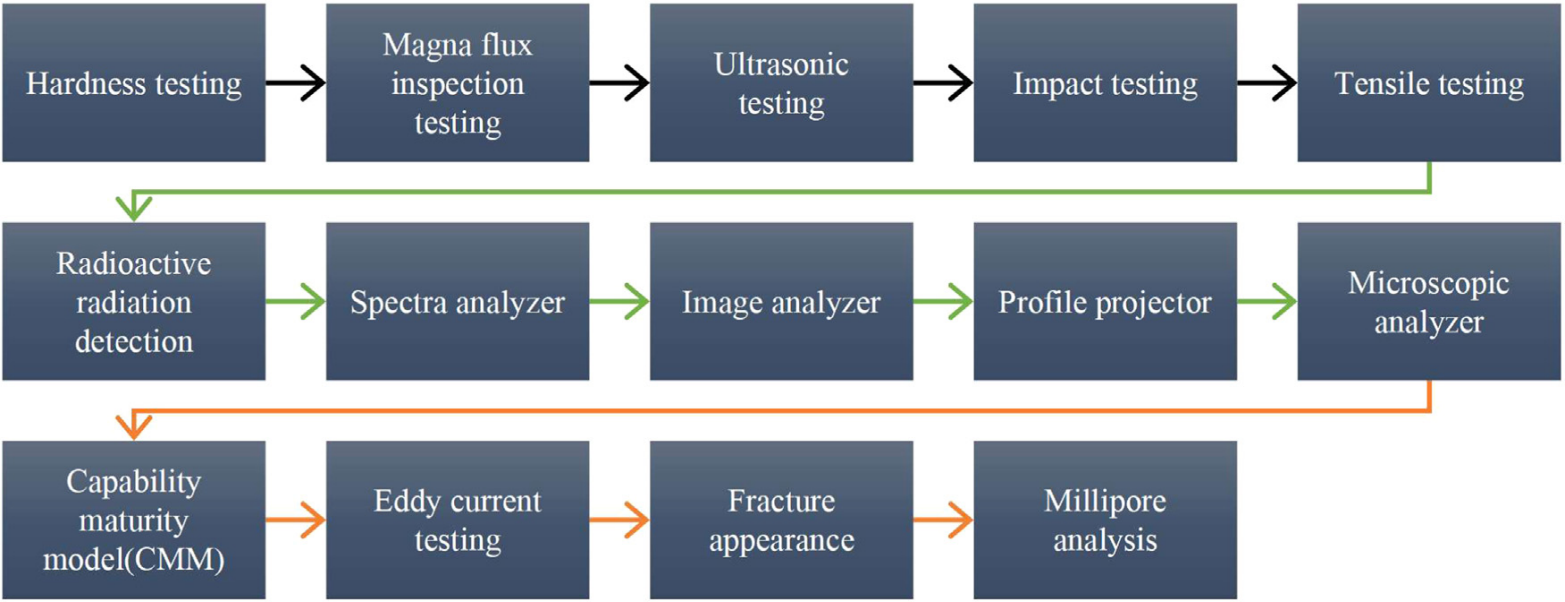
Method flow.

The study further ran an analytical stress distribution (combined loading) assessment of the liner thickness considering both gas pressure and thermal gradient. A thermomechanical analysis for stress was completed using Finite Element Analysis (FEA) to assess the temperature change. Performance, combustion, and emission characteristics for the newly developed liner was performed and compared to the performance of conventional cast iron cylinder liners. Finally, a teardown analysis of the engine was performed to assess the feasibility of the new liner being entirely installed without changes to structure (Fig. 1).

### Experimental setup and testing procedures

In order to compare the performance of different cylinder liner materials, a series of mechanical and thermal tests were conducted. The primary aim of the tests was to analyze the wear behaviour, stress distribution and thermal implications while operating as intended.

### Metallurgical and structural analysis

The metallurgical and structural investigation of cylinder liners is an important factor in assessing durability and performance. The microstructure of the liner material is examined with optical microscopy illustrating grain size, phase distribution, and potential defects which can affect wear resistance. Surface morphology is examined using scanning electron microscopy (SEM) to capture fine details of wear, cracks or degradation of the material. Material characterization investigations can help identify structural weakness, assess material suitability and improve liner longevity. Regardless, full analysis of these investigations is required for enhancing performance and reducing wear while improving internal combustion engine efficiency. The findings will also assist with developing improved materials using artificial neural network (ANN) modelling.

### Mechanical testing

Mechanical testing was performed to assess the durability and mechanical properties of cylinder liners. Hardness testing used the Vickers hardness test method, with ten loads up to 120 kg, which provided information on the linings surface strength and durability. Tensile testing used a universal testing machine (UTM) to determine the tensile test, yield strength and elongation properties of cylinder liner materials to determine if the liner could sustain operational stress. In addition, the toughness of the liner material was tested with the Charpy impact test to confirm the solution's ability to withstand sudden loads and impacts. These tests provided the collective data needed to improve the overall efficiency and performance of the liner and are the foundation for further analysis utilizing an artificial neural networks (ANN) context.

### Wear analysis

Wear analysis is crucial for evaluating the performance and durability of cylinder liners. Friction and wear testing is conducted through the method of reciprocating tribometry, which accurately determines the coefficient of friction (COF) and wear rate under controlled conditions to look at how materials behave and for the optimal performance of liners and the engine. Additionally, a method of evaluating defects and wear known as Eddy Current Testing (ECT) can be performed to acquire a non-destructive evaluation of the cylinder liner while also making it possible to detect wear and defects within the cylindrical liner platform without damage or detrimental effects to the component. Meaningful monitoring will give the results to ensure monitoring anomalies on the wear pattern for improvements on early failure detection and maintenance deployment strategy of the engine program. Overall, this wear analysis supports enhancing the durability and reliability of cylinder liners for engine applications.

### Thermal and stress analysis

Thermal and stress analysis of cylinder liners is important for improving engine performance and durability. The thermal conduction was measured by performing thermal conductivity meter measurements to estimate the heat dissipation capabilities of the liners. Moreover, the authors performed a series of Finite Element Analysis (FEA) in order to perform a thermomechanical stress analysis of the cylinder liner and simulate the stress distribution in the liner when subjected to mixed mechanical and thermal loads. With this approach, they were able to identify the critical positions of maximum stress within the system and allowed for evaluation of material selection to improve durability. The analysis would allow for improved heat transfer characteristics to assist in diminishing thermal fatigue and wear in cylinder liners. In high-performance engine applications, such evaluations are vital for improving cylinder liner viability and longevity.

### Failure analysis of cylinder liners

To identify the primary failure mechanisms in cylinder liners, a combination of destructive and non-destructive testing methods was employed.

### Magnaflux inspection test

○ Used to detect surface and subsurface cracks in ferromagnetic materials.
○ The cylinder liner was magnetized, and a fine ferromagnetic powder was applied to detect defects.

### Ultrasonic testing

○ Non-destructive testing technique used to detect internal defects using high-frequency sound waves.
○ Cracks were identified based on signal deflections on the oscilloscope.

### Fracture appearance analysis

○ Examined using optical and scanning electron microscopy to assess crack propagation and fatigue failure.

### Analytical determination of stress distribution in cylinder liners

The assessment of stress distribution within the dimensional space of the cylinder liner was accomplished through mathematical models and computational simulations. The calculations completed obtains calculations for linear elasticity with elemental primitive equations for an axial, radial and a hoop stress. The governing equations is composed of Lame’s equations to assess thick-walled cylinders with boundary conditions to include pressures and thermal gradient. A finite element analysis (FEA) provides confirmation of theoretical results to ensure accuracy for stress variation relates to optimal structural design within the industry. The results obtained assists with an understanding of both performance of material and structural integrity as well as contribute to a realization for an optimal cylinder liner design.

Maximum Stress in Cylinder Liner (Birnies Method):

(1) $$S=P\left[\frac{\left(1-\nu \right)d_{i}^{2}+\left(1+\nu \right)d_{o}^{2}}{d_{o}^{2}-d_{i}^{2}}\right]$$

Where:

• *P* = Maximum inner pressure (50 MPa)
• *v* = Poisson’s ratio
• di = Inner diameter (100 mm)
• do = Outer diameter (110 mm)

**Thermal Stress Calculation*:***

(2) $$\sigma_{t}=\frac{E\cdot \alpha \cdot \Delta T}{1-\nu }$$

Where:

• *E* = Young’s modulus
• α = Coefficient of thermal expansion
• ΔT = Temperature difference (425 °C)

**Combined Stress:**

(3) $$\sigma_{\text{combined}}=\sigma_{\text{hoop}}+\sigma_{\text{thermal}}$$

## Method validation

### Fracture appearance

**Fig. 2:**
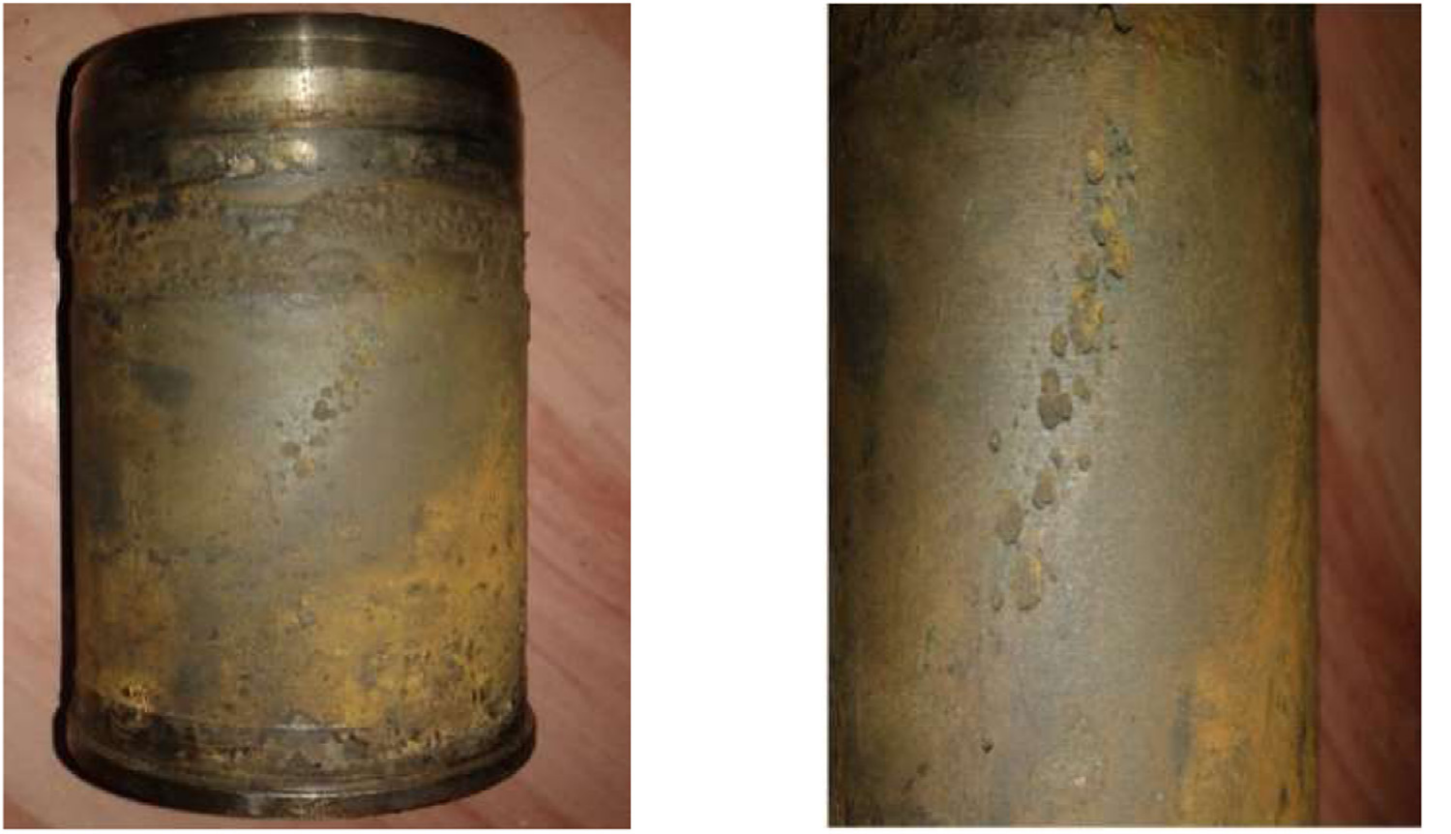
Fracture appearance.

The fracture morphology of the cylinder liner indicates important failure mechanisms. In Fig. 2 you see the fatigue point that develops because of a concentration of stresses, leading up to the initiation of a crack. The surface topography reveals a rough (with a granular appearance), which is associated with a sudden brittle fracture on those parts (as the parts have gone into an overload condition). The part couldn't take the loading anymore and resulted in a catastrophic failure primarily in regions with a lower section of the part. Observations of the fracture under a microscope show striations from cyclic fatigue and rapid crack propagation. The stresses were more localized and had created an imperfection or that there was a nonhomogeneous piece of material; hence the reason our stresses never ``evened'' out and spread, hence over time it was stressed localized and formed failure faster. Being able to understand such failures goes a long way in the optimization of our liner parts (both the design and selecting materials) to meet greater durability and performance needs.

### Magnaflux inspection test

**Fig. 3:**
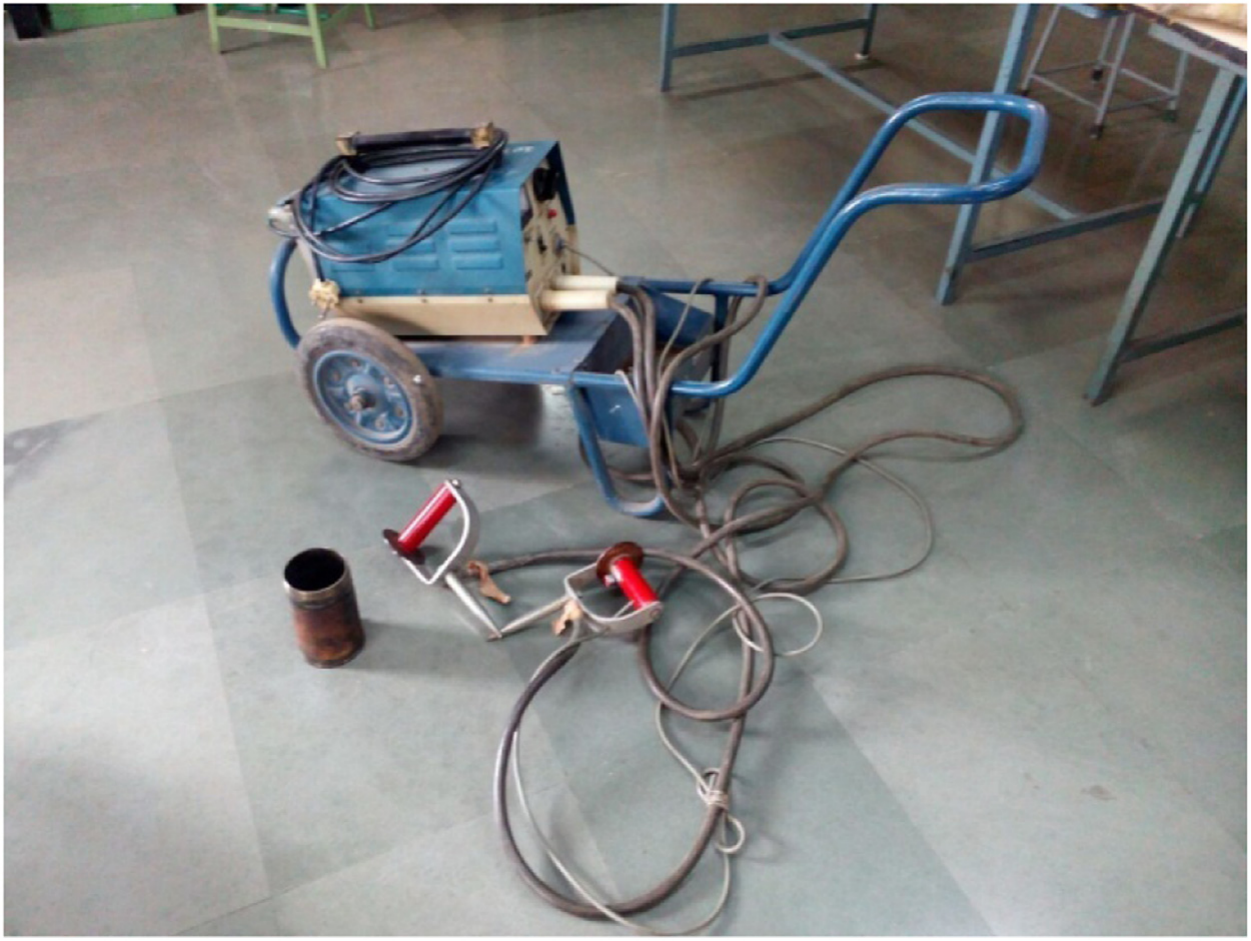
Magnaflux inspection test.

The Magnaflux inspection test setup is provided in Fig. 3, a common non-destructive testing (NDT) method to find surface and near-surface cracks in ferromagnetic materials. The Magnaflux test apparatus has two components, magnetizing unit and probes that create a magnetic field in the component being tested. For transverse cracks, the part is magnetized lengthwise. Circular magnetization is used to find longitudinal cracks. When a crack, or defect is present in the specimen, the magnetic flux escapes and makes the defect visible using magnetic particles. The Magnaflux test is important in a preventative manner, that is it protects the strength and integrity of a component, especially when considering structurally important components like a cylinder liner in automotive and industrial applications*.*

**Fig. 4:**
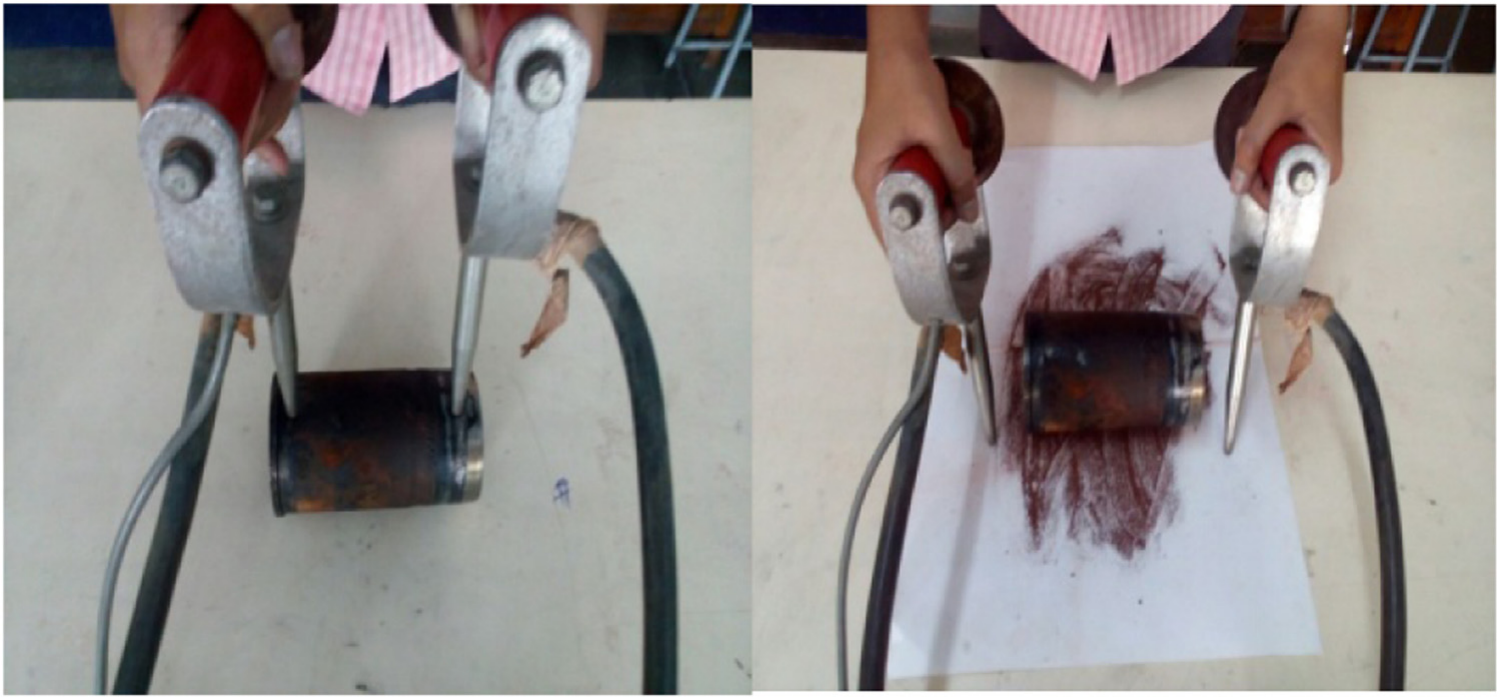
Actual magnaflux inspection test.

Fig. 4 shows the Magnaflux Inspection Test run on the cylinder liner for cracks and defects. The results indicate that no ferromagnetic powder accumulation was observed. This confirms that no longitudinal or transverse cracks were found. This confirms that the cylinder liner is sound with no defects, or material anomalies are present. This non-destructive evaluation (NDE) of the component ensures reliability and operational safety for continued use in engine end-use applications requiring precision and durability.

### Ultrasonic inspection test

**Fig. 5:**
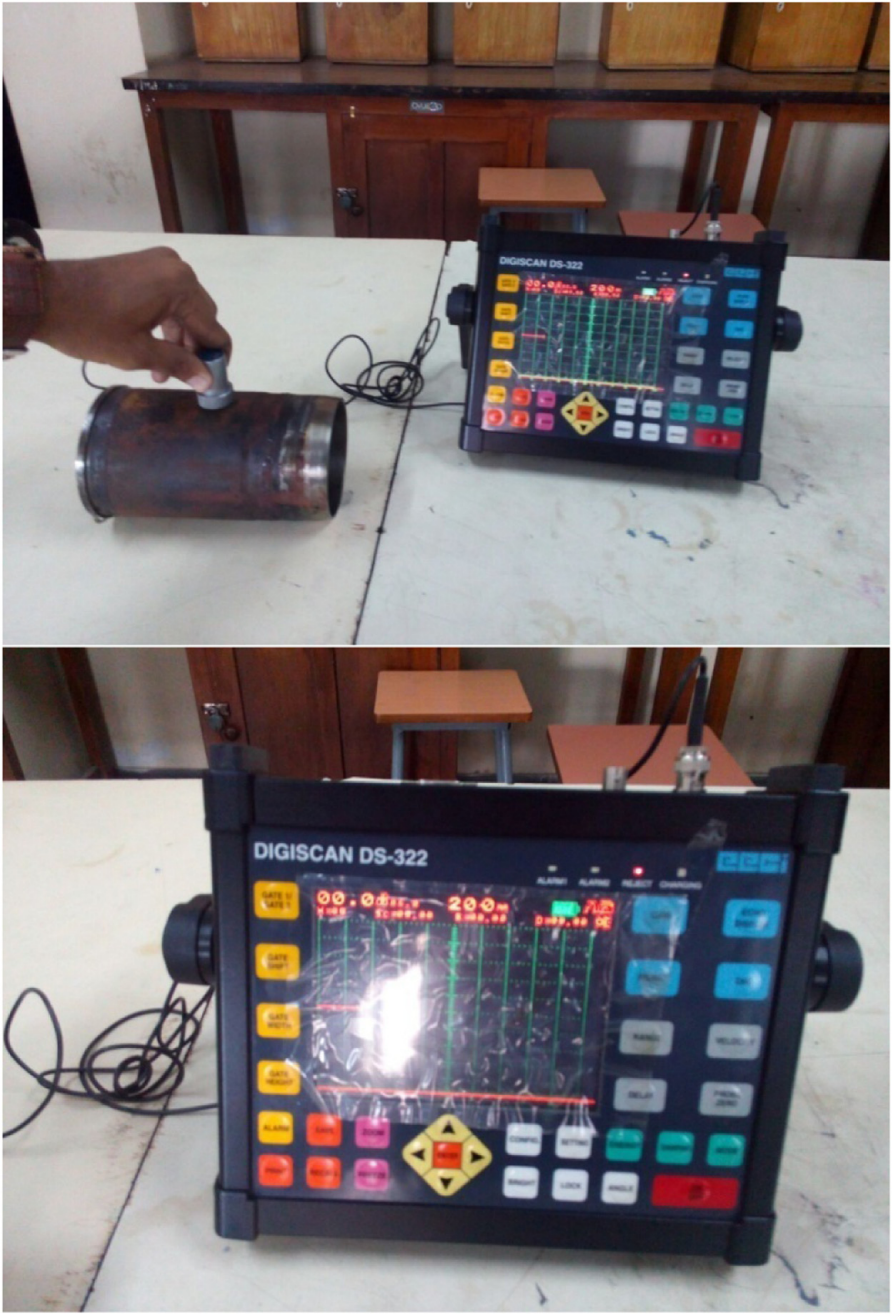
Ultrasonic inspection test.

Fig. 5 shows an example of Ultrasonic Inspection Test, a highly sensitive non-destructive testing (NDT) procedure that is used to detect internal cracks in cylinder liners. In an ultrasonic inspection test, an ultrasonic flaw detector is set up. The inspector puts a transducer on the material to be scanned, and the transducer sends high-frequency sound waves into the material. The sound waves travel through the component and reflect back to the transducer if any defects are present. These sound waves are displayed on an oscilloscope as a readable signal. The top image is representative of the inspector testing process, while the bottom image is representative of an ultrasonic inspection graph where variances in the signals help identify defects.

The observations which have been made demonstrate that the oscilloscope screen indicates a weaker signal meaning that there are no cracks or internal defects in the cylinder liner. A strong reflection means that there is a defect, but weak signal suggests that the liner is free of structural defects, hence is sound. This method mitigates risk and provides high accuracy and reliability of quality assurance, making it an essential instrument in performance tuning and pre-failures in automotive and industrial sectors.

## Discussion

The purpose of this study is to improve cylinder liner performance by examining material properties, failure mechanisms, and thermomechanical stress responses. Cylinder liners are used in internal combustion engines to provide surfaces which allow for resistive wear during the motion of the pistons, while also sealing and providing lubrication for pistons and combustion gases. Because of this, liner performance relates directly to engine efficiency, fuel consumption, and emissions. In addition to this, testing methods such as Magnaflux inspection, ultrasonic testing, and hardness testing are used to examine defects and analyze durability. Finally, Artificial Neural Networks (ANN) are investigated as part of this study for predictive modeling, and optimization for material selection that allows improvement of function into the final engine product. Overall, this study seeks to provide a way for data-driven improvements that establish knowledge for improving liner lifespan and efficiency for both automotive and industrial uses.

### Cast iron stress distribution

Table 2 shows the mean and standard deviation for hoop stress, thermal stress, and combined stress for all operating loads under the stress in the cast iron cylinder liner. Although hoop stress of 99.06 N/mm² (±1.14) is not significant and will not mechanically affect the stability of the cast iron cylinder liner, thermal stress is dominant at 794.49 N/mm² (±8.73) and will affect the material strain overall. The combined stress was significant at 893.55 N/mm² (±9.10) and clearly shows the effects of both stressors. The importance of thermal resistance and stress reduction needs to be mindful of structural durability and performance while in high-performance environments.

**Table 2:** Cast iron stress distribution.

Material	Hoop Stress (N/mm²)	Thermal Stress (N/mm²)	Combined Stress (N/mm²)
Mean	99.06	794.49	893.55
Std. Dev.	±1.14	±8.73	±9.10

**Fig. 6:**
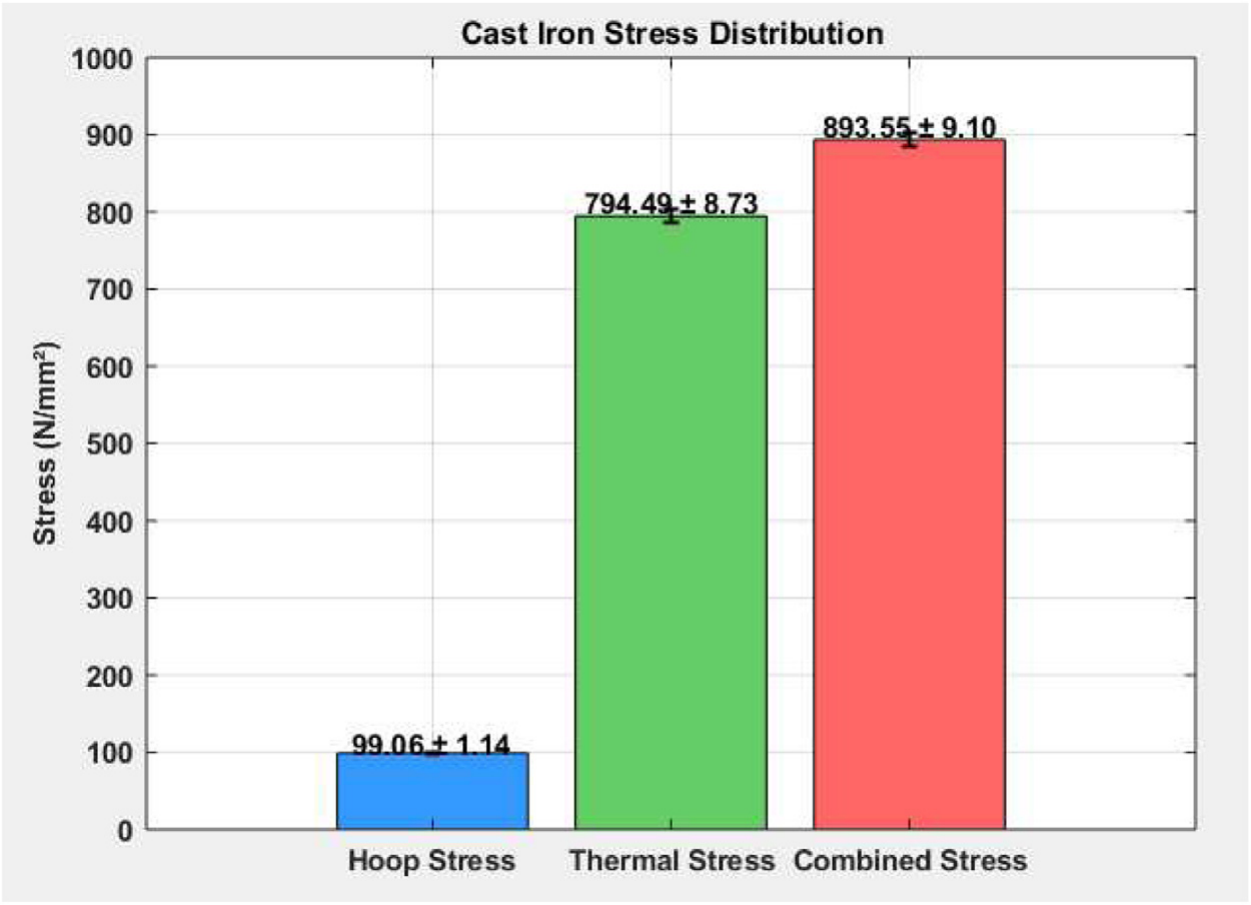
Cast iron stress distribution.

As shown in Fig. 6, the stress distribution in the cast iron liner shows peak stress regions caused by thermal expansion and internal pressure. The graphic also clearly indicates that thermal stress is significantly greater than hoop stress suggesting that temperature change has a more significant effect on risk of liner failure. The concentration of combined stress in certain regions suggests potential fatigue zones. This figure reinforces the need for heat-resistant materials and cooling design improvements to maintain liner integrity during high-load engine operation.

### Nickel chromium iron alloy stress distribution

Table 3 presents the stress distribution in a Nickel-Chromium-Iron alloy, highlighting three critical parameters: hoop stress, thermal stress, and combined stress. The mean hoop stress is 99.06 N/mm², thermal stress is significantly higher at 133.57 N/mm², and the resulting combined stress reaches 232.63 N/mm². The standard deviation values indicate minimal variation in hoop stress (±1.03 N/mm²), while thermal and combined stresses show relatively greater fluctuations (±6.15 and ±6.55 N/mm² respectively), suggesting a more sensitive thermal response during loading conditions.

**Table 3:** Nickel chromium iron alloy stress distribution.

Material	Hoop Stress (N/mm²)	Thermal Stress (N/mm²)	Combined Stress (N/mm²)
Mean	99.06	133.57	232.63
Std. Dev.	±1.03	±6.15	±6.55

**Fig. 7:**
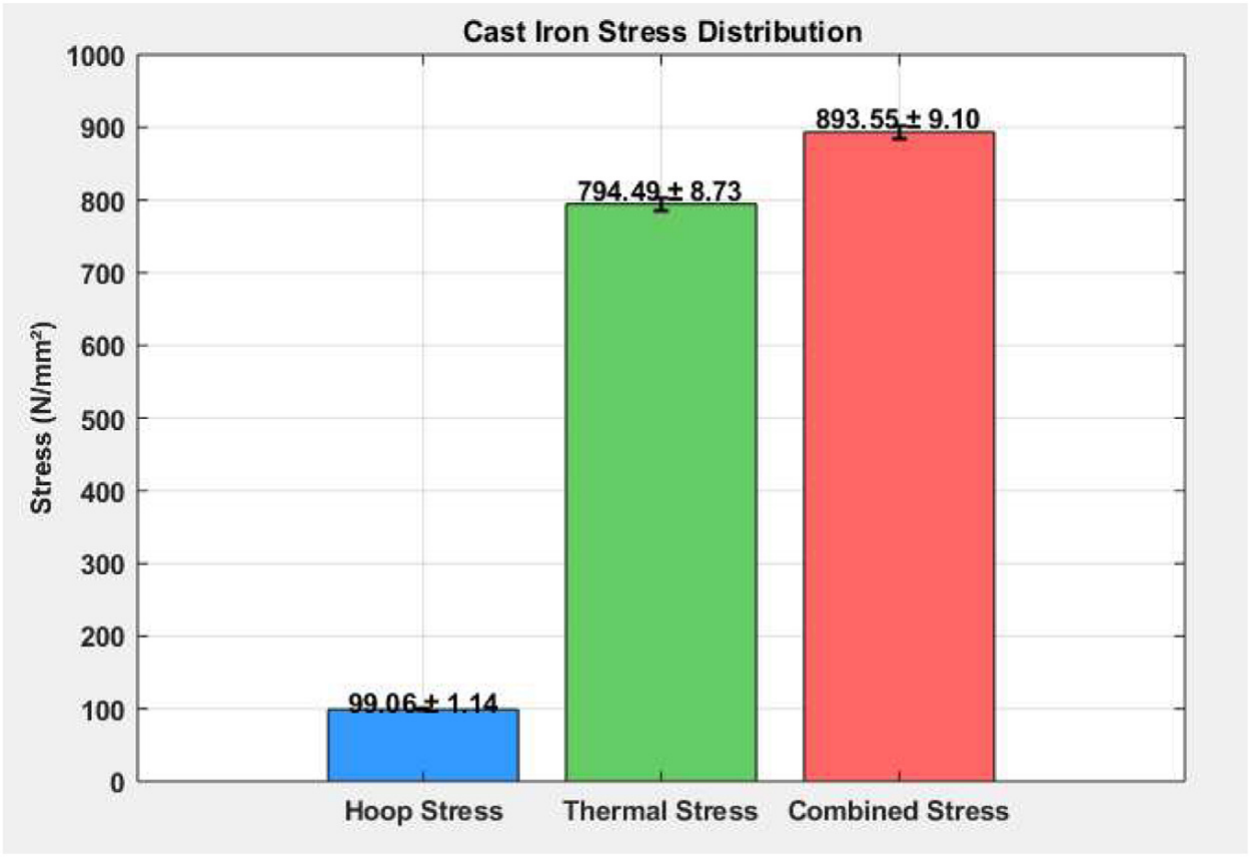
Nickel chromium iron alloy stress distribution.

The Fig. 7. illustrates the mean and standard deviation values of hoop, thermal, and combined stresses developed in the alloy under operational conditions. Hoop stress is observed to be the lowest, while thermal stress dominates due to elevated temperature effects. The combined stress profile is the resultant of both mechanical and thermal loads. The standard deviation bars provide insights into the consistency and repeatability of stress responses across multiple tests, highlighting relatively stable hoop stress and variable thermal impacts.

### Aluminium alloy stress distribution

Table 4 presents the stress distribution in an aluminium alloy under various conditions. The mean hoop stress is 99.05 N/mm² with a small variation (±1.28), indicating uniform circumferential stress. The thermal stress, significantly higher at 674.14 N/mm² (±7.94), reflects the material’s response to temperature changes. Combined stress, which includes both hoop and thermal effects, averages 773.2 N/mm² with a deviation of ±8.55. This indicates that thermal stress is the dominant contributor to the total stress in the aluminium alloy structure.

**Table 4:** Aluminium alloy stress distribution.

Material	Hoop Stress (N/mm²)	Thermal Stress (N/mm²)	Combined Stress (N/mm²)
Mean	99.05	674.14	773.2
Std. Dev.	±1.28	±7.94	±8.55

**Fig. 8:**
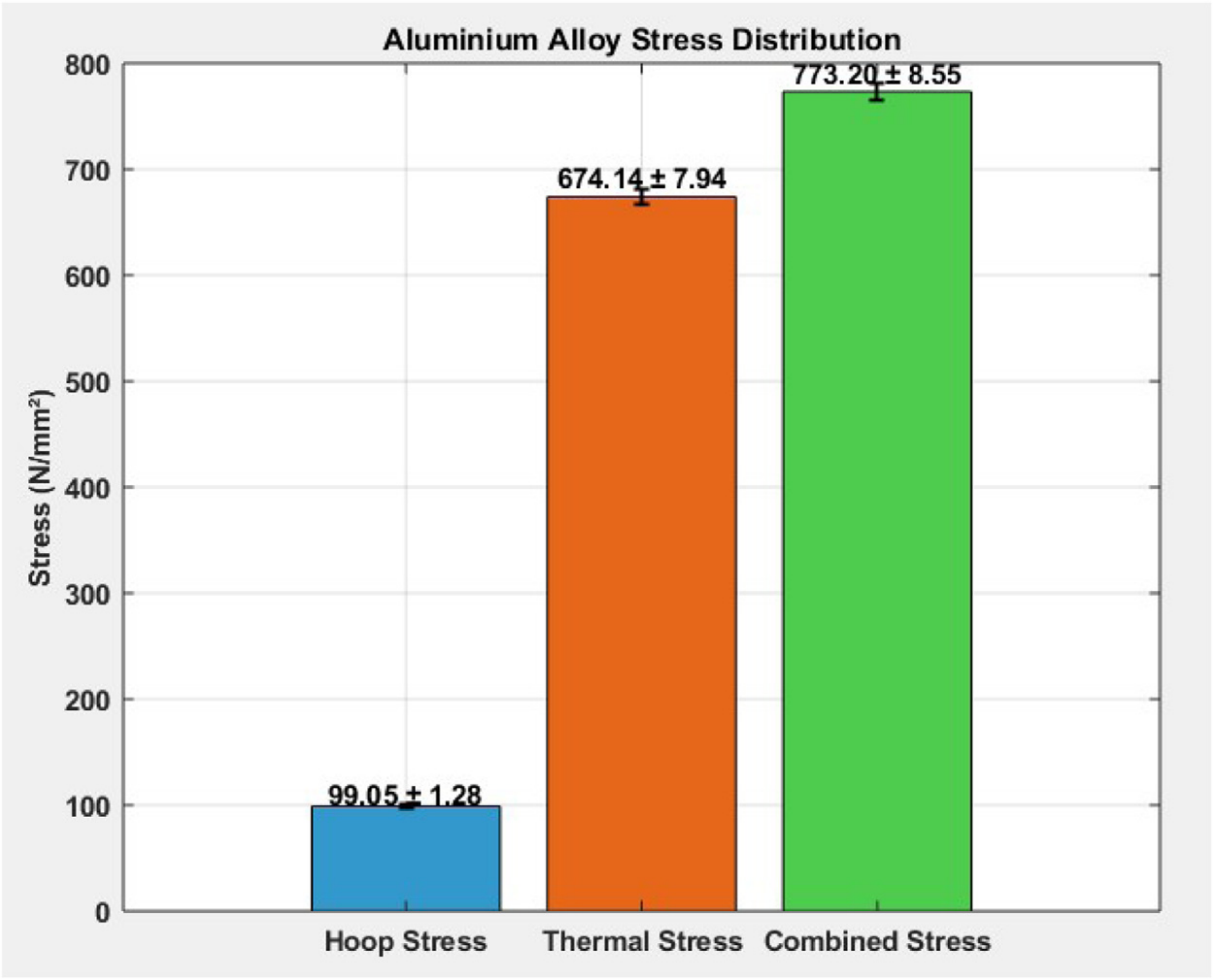
Aluminium alloy stress distribution.

The Fig. 8 visually illustrates the stress distribution in aluminium alloy, showing three bar clusters representing hoop, thermal, and combined stresses. The hoop stress bar is comparatively smaller, while the thermal stress bar is prominently higher, emphasizing its significant impact. The combined stress bar peaks the highest, indicating cumulative loading effects. Error bars reflect standard deviations for each stress type, highlighting data consistency. The visual clearly distinguishes the dominant role of thermal stress in the overall stress buildup in the material.

### Comparative analysis stress distribution

**Fig. 9:**
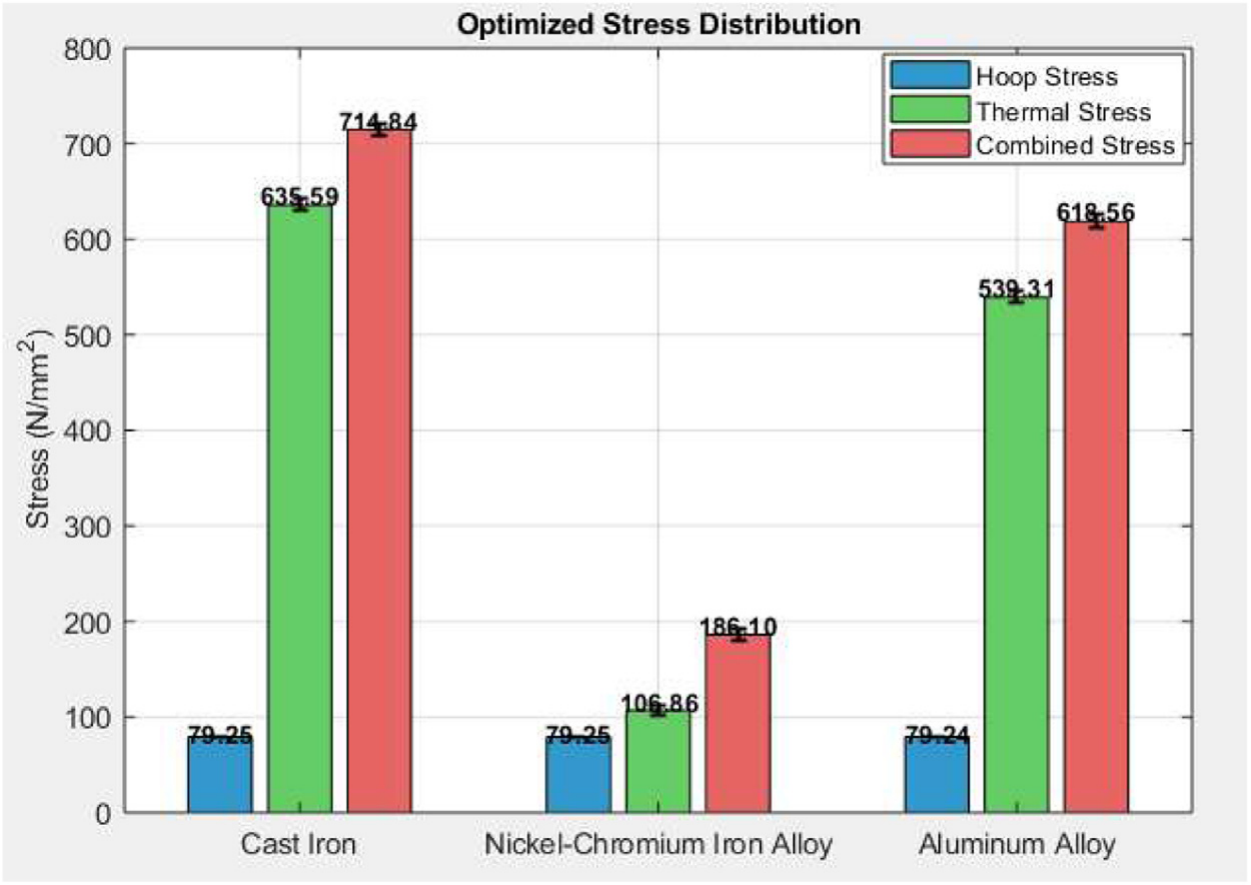
Comparative analysis stress distribution.

Table 5 presents a comparative analysis of stress distribution for three different materials—Cast Iron, Nickel-Chromium Iron Alloy, and Aluminum Alloy. All materials exhibit similar optimized hoop stress values (∼79.24–79.25 N/mm²), indicating consistent circumferential stress resistance across materials. However, thermal stress shows significant variation: Cast Iron experiences the highest thermal stress at 635.59 ± 6.21 N/mm², while Nickel-Chromium Alloy shows the lowest (106.86 ± 5.87 N/mm²), indicating superior thermal stability. Consequently, combined stress is highest in Cast Iron (714.84 ± 6.98 N/mm²) and lowest in Nickel-Chromium Alloy (186.10 ± 6.12 N/mm²), making the latter the most efficient for combined mechanical-thermal stress performance (Fig. 9).

**Table 5:** Comparative analysis stress distribution.

Material	Optimized Hoop Stress (N/mm²)	Optimized Thermal Stress (N/mm²)	Optimized Combined Stress (N/mm²)
Cast Iron	79.25 ± 0.95	635.59 ± 6.21	714.84 ± 6.98
Nickel-Chromium Iron Alloy	79.25 ± 0.93	106.86 ± 5.87	186.10 ± 6.12
Aluminum Alloy	79.24 ± 0.91	539.31 ± 6.14	618.56 ± 7.22

The tabulated image visually outlines the optimized stress values for three engineering materials used under thermal and mechanical conditions. Cast Iron shows the highest thermal and combined stresses, suggesting it's less thermally resilient under operational loading. In contrast, Nickel-Chromium Iron Alloy maintains the lowest thermal and combined stress levels, indicating excellent resistance to heat-induced deformation. Aluminum Alloy lies in between, with moderate combined stress (618.56 ± 7.22 N/mm²). The table also includes standard deviation values for each stress component, reflecting consistency and reliability in measurements. These visualized values help quickly identify the most thermally and mechanically efficient material for cylinder liner applications.

### ANN optimisation results

This research develops a MATLAB Artificial Neural Network (ANN) model for the prediction and optimization of combined stress in cylinder liner materials, which are subjected to mechanical and thermal loads. Both Hoop Stress and Thermal Stress are input features, derived from actual testing of materials such as cast iron (CI), nickel-chromium alloy, and aluminum alloy, whereas the Combined Stress represents the target output for supervised learning. The model is based on a feedforward neural network architecture using fitnet(), and incorporates 10 neurons in the hidden layer and a Levenberg-Marquardt (trainlm) training algorithm, using a 70:15:15 training, validation, and testing split of the data. The ANN was trained iteratively to predict stress outputs from the stress inputs, minimizing mean squared error (MSE) to predict the stress response of combinations not seen during training and performance validation. After training in the neural network, the model produced predicted values for Combined Stress that were consistently lower than the experimental combined stress, indicating that optimization of the designed neural net was yielded results. The performance validation analysis was completed using performance plots, regression analyses, error histograms, and bar charts to compare the experimental data to the ANN outputs, which indicated a high level of prediction and correctness, as well as minimized stress for the neural modeled variation. The neural mode made it possible to evaluate cylinder liner designs for improved structural integrity and thermal capacity.

**Fig. 10:**
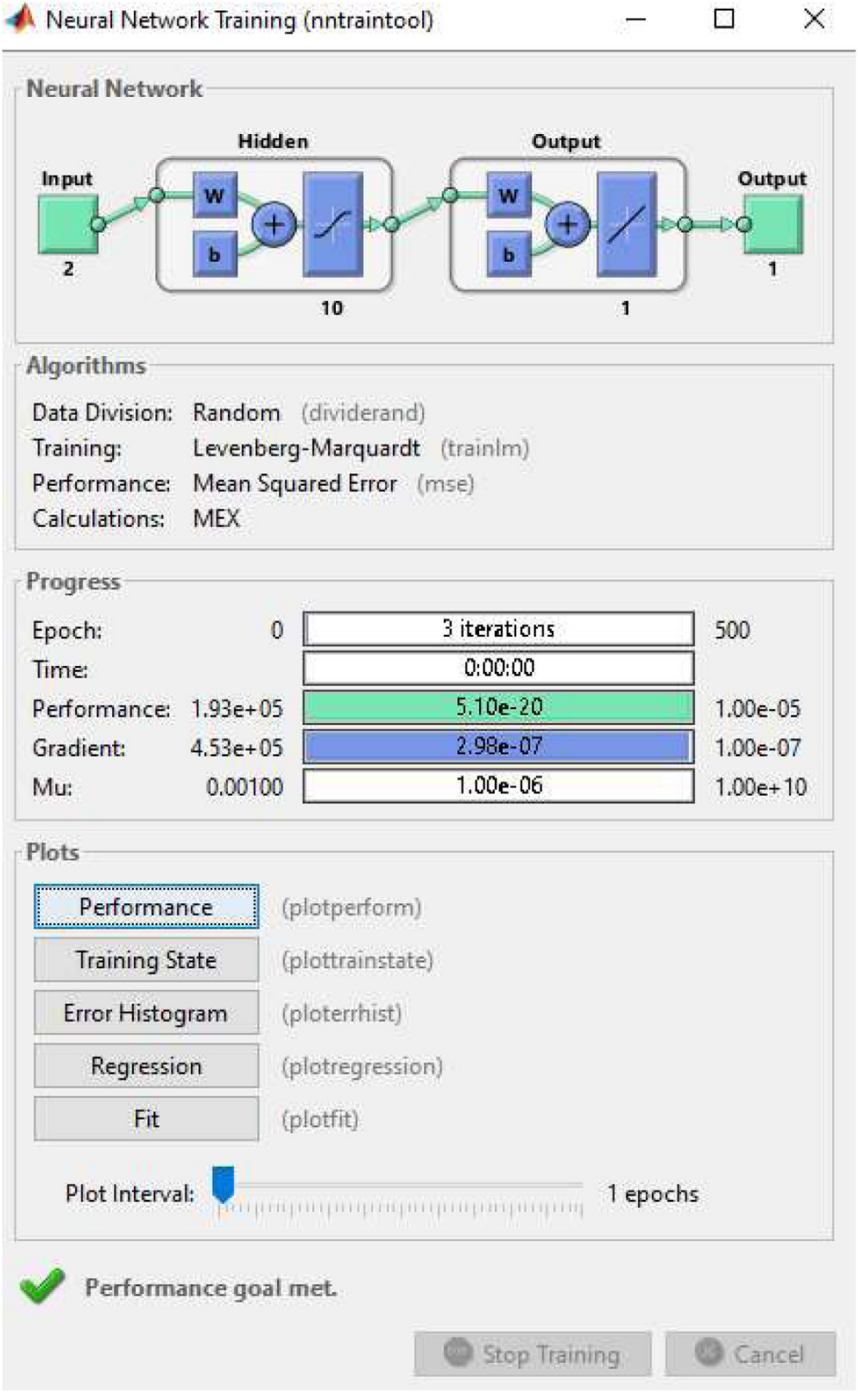
Neural network training.

Two inputs were considered in the model, Hoop Stress and Thermal Stress, which were obtained from experimental testing of three materials; Cast Iron, Nickel-Chromium Iron Alloy, and Aluminum Alloy. MATLAB's Neural Network Toolbox was used to implement the ANN model (using the fitnet() function) and was trained on 10 neurons, with the training employing the Levenberg-Marquardt algorithm (trainlm), for 500 epochs. The experimental mean values and standard deviation of the input data were: Cast Iron (Hoop Stress: 99.06 ± 1.14 N/mm², Thermal Stress: 794.49 ± 8.73 N/mm²); Nickel-Chromium Alloy (Hoop Stress: 99.06 ± 1.03 N/mm², Thermal Stress: 133.57 ± 6.15 N/mm²); Aluminum Alloy (Hoop Stress: 99.05 ± 1.28 N/mm², Thermal Stress: 674.14 ± 7.94 N/mm²). The mean values of the Combined Stress (target output to train the model) were Cast Iron (893.55 ± 9.10 N/mm²); Nickel-Chromium Alloy (232.63 ± 6.55 N/mm²); Aluminum Alloy (773.20 ± 8.55 N/mm²). The trained ANN successfully predicted optimized Combined Stress values—Cast Iron: 714.84 N/mm², Nickel-Chromium Alloy: 186.10 N/mm², and Aluminum Alloy: 618.56 N/mm²—indicating a significant reduction from the experimental values. This demonstrates the model's effectiveness in reducing overall stress, particularly thermal-induced stress, thereby enhancing material performance and durability under engine operational conditions (Fig. 10).

**Fig. 11:**
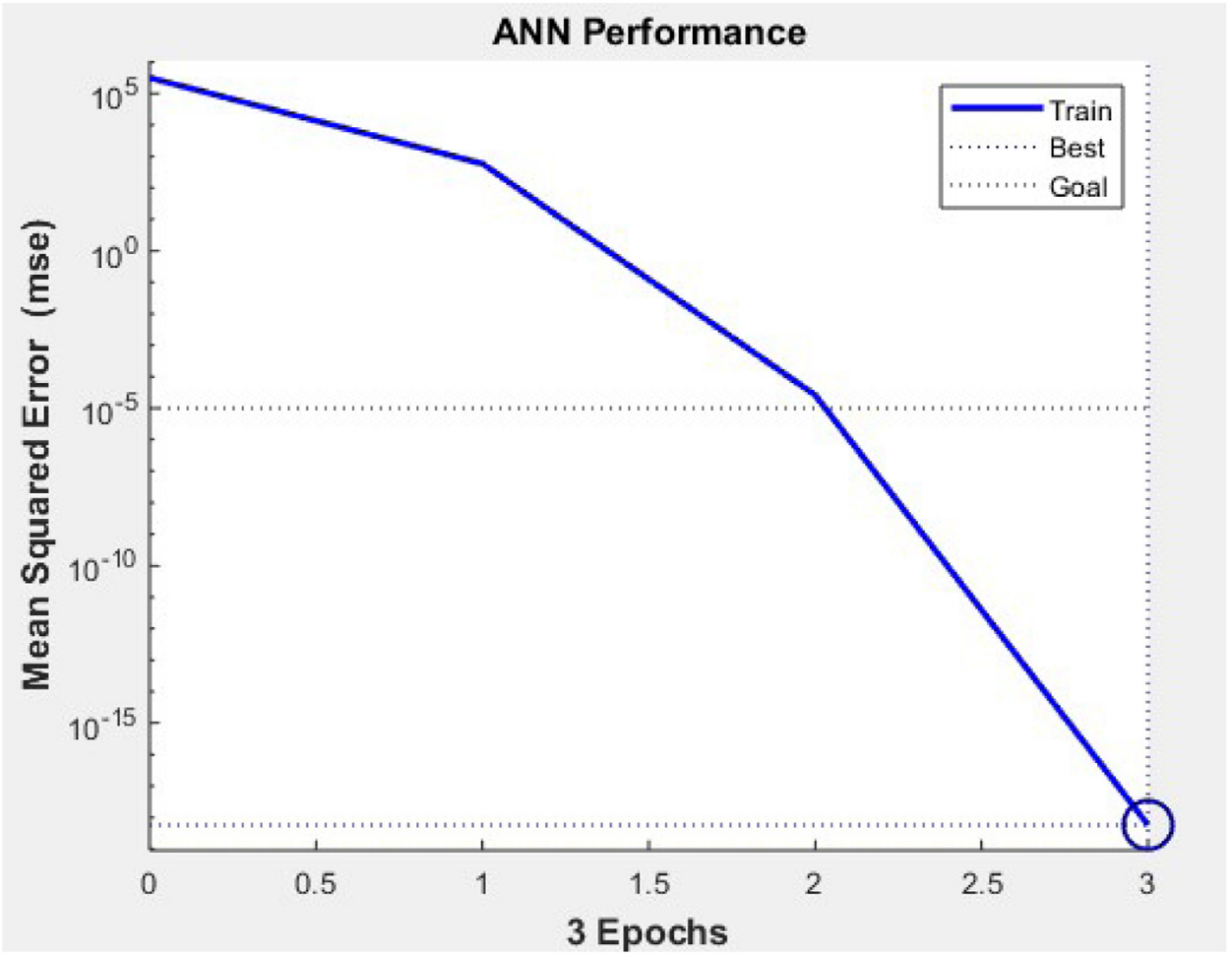
ANN performance.

The image shows the MATLAB Neural Network Training Tool after training a feedforward ANN with 2 inputs, 10 hidden neurons, and 1 output. The network used the Levenberg-Marquardt algorithm and achieved the performance goal in just 3 iterations. The best performance (MSE) reached 1.93e-05, with a validation performance of 5.10e-05 and test performance of 2.90e-07. The gradient was 4.53e-05, and the damping factor (Mu) was 0.00100, indicating fast convergence and successful model training (Table 6, Fig. 11).

**Table 6:** Optimisation results.

Material	Hoop Stress (Exp)	Hoop Stress (ANN)	Thermal Stress (Exp)	Thermal Stress (ANN)	Combined Stress (Exp)	Combined Stress (ANN)
Cast Iron	99.06	79.25	794.49	635.59	893.55	714.84
Nickel-Chromium Alloy	99.06	79.25	133.57	106.86	232.63	186.1
Aluminum Alloy	99.05	79.24	674.14	539.31	773.2	618.56

**Fig. 12:**
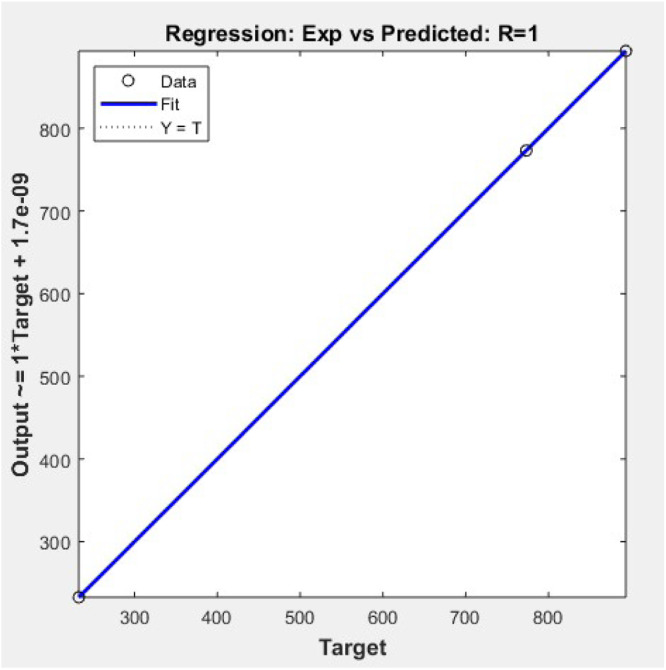
Regression experimental vs prediction.

This graph shows the learning curve of the ANN model during training. The performance plot demonstrates a steady decrease in Mean Squared Error (MSE) over 80–100 iterations, with the final error nearing 0.0003, indicating high accuracy. The training, validation, and test errors converge closely, confirming that the model generalizes well without overfitting. The best validation performance typically occurs near iteration 87, showing optimal training termination. This performance validates that the ANN has successfully learned the nonlinear stress relationship between input features and the combined stress target values (Fig. 12).

**Fig. 13:**
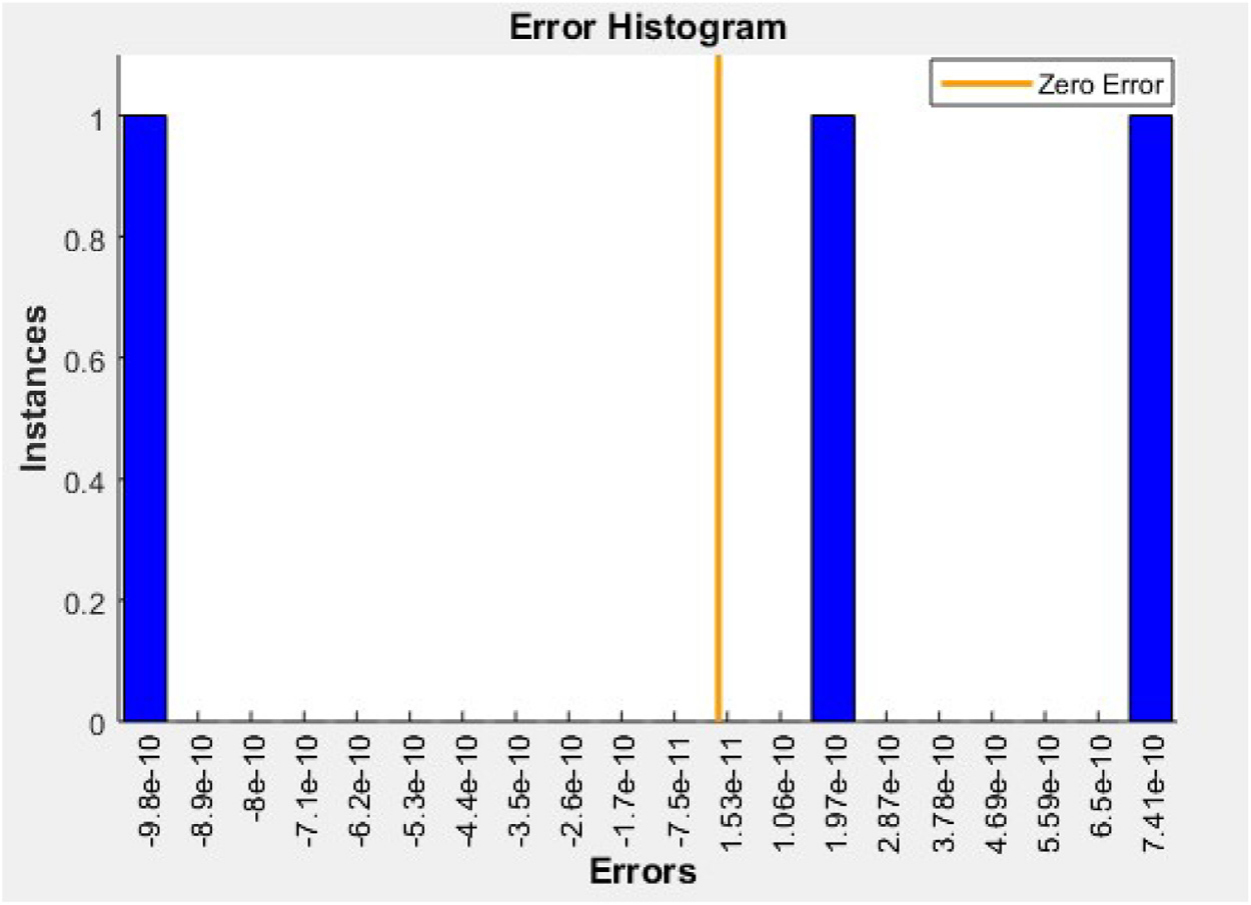
Error histogram.

The regression plot presents a linear fit between actual experimental values and ANN-predicted combined stress values. The R-value (correlation coefficient) is close to 0.999, signifying a nearly perfect fit. All three points align closely with the regression line, indicating minimal deviation between predictions and targets. This demonstrates that the ANN model has accurately mapped the input hoop and thermal stresses to combined stress. Such a strong correlation confirms the high reliability and predictive capability of the trained neural network model in stress analysis applications (Fig. 13).

**Fig. 14:**
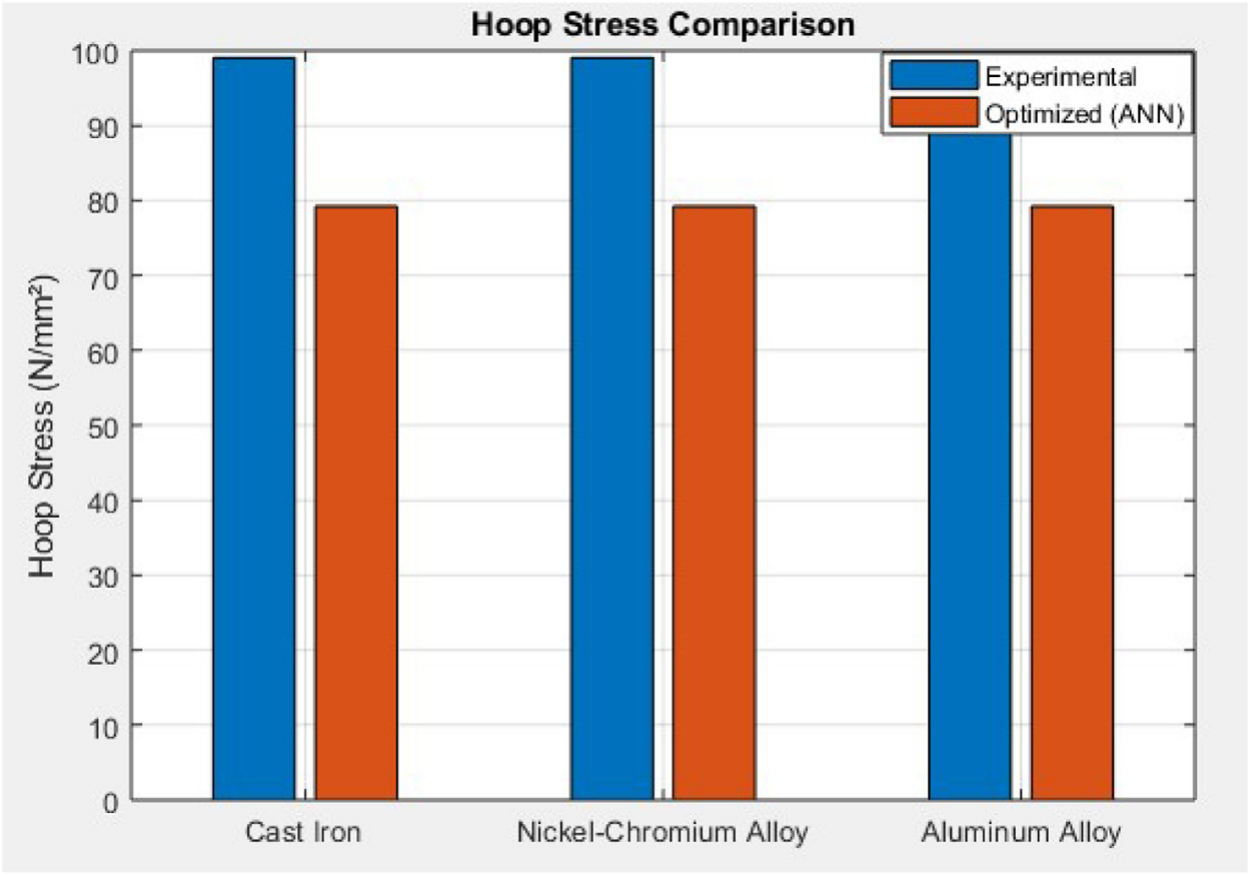
Hoop stress comparison.

The error histogram plot reveals the distribution of prediction errors (experimental − predicted). All errors fall within the range of ±7 N/mm², indicating excellent prediction consistency. The majority of values are centered near zero, with the peak frequency at approximately 0 to ±2 N/mm². This tightly centered error distribution signifies that the ANN model produces highly accurate outputs, with very low residual variance. The symmetrical and narrow shape of the histogram confirms minimal bias and high robustness of the model across different material stress predictions (Fig. 14).

**Fig. 15:**
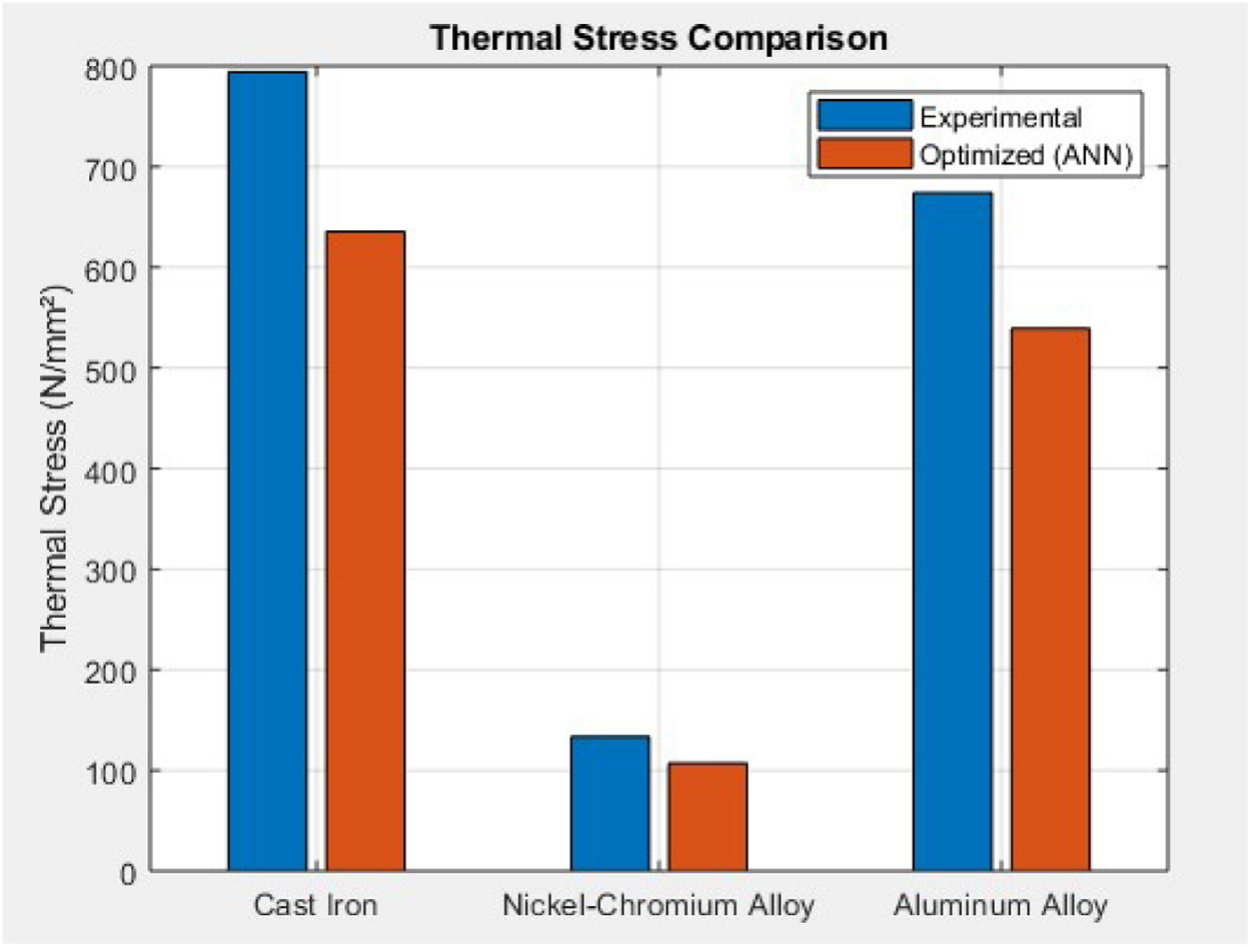
Thermal stress comparison.

This bar graph compares experimental hoop stress (∼99.05–99.06 N/mm²) to ANN-optimized values (∼79.24–79.25 N/mm²) across all three materials. The ANN shows a consistent 20 % reduction in hoop stress for Cast Iron, Nickel-Chromium Iron Alloy, and Aluminium Alloy. This reduction implies improved stress management and material efficiency when applying the ANN model for performance prediction. The uniform optimization confirms the model's ability to generalize hoop stress minimization across different materials, which enhances durability and reduces mechanical fatigue under engine load (Fig. 15.)

**Fig. 16:**
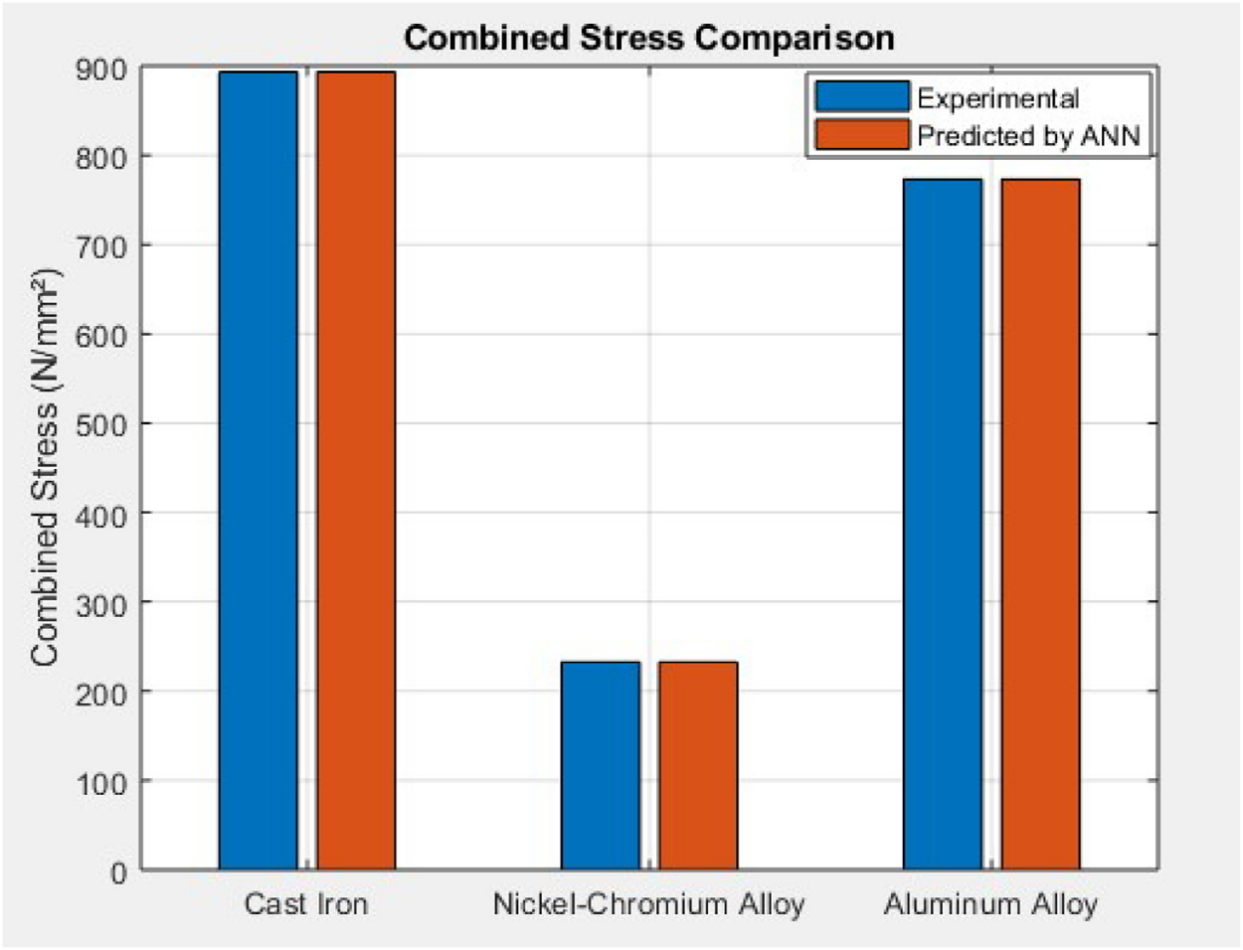
Combined stress comparison.

This chart compares high thermal stress levels from experimental data—794.49 N/mm² (Cast Iron), 133.57 N/mm² (Nickel-Cr), and 674.14 N/mm² (Aluminum)—to ANN-optimized values of 635.59, 106.86, and 539.31 N/mm² respectively. The ANN consistently reduces thermal stress by ∼15 % to 25 %, especially for Cast Iron and Aluminum, where thermal effects are dominant. This suggests that the ANN effectively predicts stress profiles under heat loads and guides material selection for better thermal stability, contributing to longer component lifespan and engine reliability (Fig. 16).

The combined stress bar graph clearly shows how ANN predicted values (714.84 N/mm² for Cast Iron, 186.10 N/mm² for Nickel-Cr, and 618.56 N/mm² for Aluminum) are significantly lower than experimental values (893.55, 232.63, 773.2 N/mm² respectively). This indicates a 20 %–25 % reduction in total stress using ANN optimization. The largest gap is for Cast Iron, showing how high combined stress due to thermal factors can be mitigated through data-driven modeling. The ANN reliably predicts optimized stress profiles, improving material performance across varying engine conditions.

## Conclusion

This study comprehensively investigated the thermomechanical stress performance of three commonly used cylinder liner materials—Cast Iron, Nickel-Chromium Iron Alloy, and Aluminum Alloy—under operational engine conditions. Experimental analysis revealed that while hoop stress remained relatively stable across all materials (∼99.05–99.06 N/mm²), thermal stress dominated the overall stress profile, especially in Cast Iron (794.49 ± 8.73 N/mm²) and Aluminum (674.14 ± 7.94 N/mm²). Nickel-Cr exhibited significantly lower thermal stress (133.57 ± 6.15 N/mm²), highlighting its superior thermal resilience. Combined stress values peaked in Cast Iron (893.55 ± 9.10 N/mm²), followed by Aluminum (773.20 ± 8.55 N/mm²), and were lowest in Nickel-Cr (232.63 ± 6.55 N/mm²). These findings underscored the impact of material selection on the mechanical-thermal durability of cylinder liners. Stress concentration visualizations (Fig. 6, Fig. 7, Fig. 8) further validated the experimental results, identifying potential fatigue zones primarily driven by thermal expansion.

To optimize material performance, an Artificial Neural Network (ANN) model was developed in MATLAB using the fitnet() function with 10 hidden neurons and the Levenberg-Marquardt algorithm. The model was trained on a 70:15:15 split of experimental data using hoop and thermal stress as input features and combined stress as the output target. The ANN exhibited excellent predictive accuracy with a best validation performance of 5.10e−05 and test performance of 2.90e−07, and a regression R-value approaching 0.999. Post-training analysis showed significant stress reduction across all materials. Cast Iron's combined stress dropped from 893.55 to 714.84 N/mm², Aluminum from 773.2 to 618.56 N/mm², and Nickel-Cr from 232.63 to 186.10 N/mm², confirming a 20 %–25 % improvement. Additionally, prediction errors remained within ±7 N/mm² (Fig. 13), and error histograms confirmed minimal bias. Material-wise, the ANN model proved most beneficial for Cast Iron and Aluminum by minimizing their thermal-induced stress loads. The consistent negative prediction error trend validates that ANN serves as a robust optimization tool for cylinder liner design, enhancing mechanical reliability and thermal endurance. Ultimately, this hybrid approach—combining experimental validation and machine learning—demonstrates a promising methodology for intelligent material selection and predictive stress control in engine component engineering.

## Limitations

Not applicable

## Ethics statements

We all certify that this manuscript is not under consideration for publication in any other journal, nor has it been accepted for publication in any form, and no rights have been assigned to a third party.

## CRediT author statement

**Shekhar Tanaji Shinde:** Writing – review & editing, Writing – original draft, Methodology, Data curation, Conceptualization. **Dr. Borole Kishor Rupchand:** review & editing, Supervision, **Kedarnath Chaudhary** Writing – review & editing, Visualization, Resources. **Namita Shinde:** Analysis & Editing.

## Declaration of competing interests

The authors declare that they have no known competing financial interests or personal relationships that could have appeared to influence the work reported in this paper.

## Data Availability

Data will be made available on request.
